# Immediate Attention Enhancement and Restoration From Interactive and Immersive Technologies: A Scoping Review

**DOI:** 10.3389/fpsyg.2020.02050

**Published:** 2020-08-19

**Authors:** Adam C. Barton, Jade Sheen, Linda K. Byrne

**Affiliations:** School of Psychology, Faculty of Health, Deakin University, Geelong, VIC, Australia

**Keywords:** attention restoration, attention enhancement, attention state training, video games, virtual reality, exergames, flow

## Abstract

Interactive and immersive technologies such as video games, exergames, and virtual reality are typically regarded as entertainment mediums. They also offer a multitude of health and well-being benefits. They have the capacity to incorporate established well-being techniques (e.g., mindfulness, exercise, and play) and expose users to beneficial environment settings with greater ease, improved access, and a broader appeal. The authors conducted a scoping review to explore whether these technologies could be used to benefit attention in healthy adults, that is, in a regulatory sense such as during periods of cognitive fatigue or attention-critical tasks. Research efforts have typically focused on long-term practice methods for attention enhancement with these technologies. Instead, this review provides the first attempt to unify a broad range of investigations concerned with their immediate impact on attention through state-change mechanisms. This applies the concept of attention state training and a growing evidence base, which suggests that meditative practices, exercise bouts, and nature exposures can provide short-term improvements in attentional performance following brief interactions. A systematic search of MEDLINE, Embase, and PsycINFO databases resulted in 11 peer-reviewed articles (13 experimental trials) each including at least one objective measure of attention directly following the use of an interactive or immersive technology. Most studies involved interactive technologies (i.e., video games and exergames), whereas there were three immersive interventions in the form of virtual reality. The comparisons between baseline and postintervention showed mostly no effect on attention, although there were five cases of improved attention. There were no instances of negative effects on attention. The results are significant considering mounting concerns that technology use could be detrimental for cognitive functioning. The positive effects reported here indicate a need to specify the type of technology in question and bring attention to positive vs. negative technology interactions. Implications for the literature concerning attention state training are discussed considering promising effects of technology exposures geared toward flow state induction. Significant gaps in the literature are identified regarding the implementation of traditional attention state training practices.

## Introduction

Attention is considered a “superior cognitive function” because it has a critical and wide-spreading influence over our daily life activities (Rueda et al., 2015). This is due to its multifaceted nature, comprised of both distinct and overlapping neural networks that provide top–down control and bottom–up supervisory functions (Norman and Shallice, 1986; Petersen and Posner, 2012; Diamond, 2013; Katsuki and Constantinidis, 2014). Top–down attention is the focus of this review. This refers to the voluntary process of allocating resources to a chosen perceptual input (Baluch and Itti, 2011); exceptionally difficult in environments filled with distractions (Wilmer et al., 2017) and when the mind wanders uncontrollably off-task (Killingsworth and Gilbert, 2010; Baldwin et al., 2017). These top–down aspects of attention are often subdivided into executive and sustained components of attention. Executive attention (which includes selective attention, directed attention, cognitive control, or attentional inhibition) is primarily linked with the ability to direct focus where desired whilst ignoring competing information (Diamond, 2013; Engelhardt et al., 2015; Rueda et al., 2015). Sustained attention (also termed vigilance or tonic attention) is the ability to maintain focus on task-relevant information for a long period of time (Oken et al., 2006; Rueda et al., 2015).

Studies have shown that top–down attentional ability is constantly fluctuating according to changes in our physical, emotional, and mental states. For instance, prolonged mental effort is associated with periods of cognitive fatigue and impaired executive attention (Ohly et al., 2016; Stevenson et al., 2018). Changes in mood have also been linked with inattention, with sadness found to be a significant precursor of mind wandering (Poerio et al., 2013) and both negative and positive affect causing increased distractibility (Pacheco-Unguetti and Parmentier, 2014, 2016). Early evidence also suggests that goal-directed, sustained attention fluctuates according to the time of day, resulting from a number of possible factors such as arousal, environmental pressure, and the human circadian rhythm (Smith et al., 2018). These fluctuations are a concern particularly during safety critical occupations (e.g., air traffic controllers, pilots, transportation security officers, medical professionals) where inattentive episodes present major safety and security risks (Petrilli et al., 2006; Rupp et al., 2017). This extends to other occupations and populations as well, with poor attention associated with negative effects across learning and education (Sedgwick, 2018; Lodge and Harrison, 2019) and health and well-being (Llewellyn et al., 2008; Livingstone and Isaacowitz, 2017). The dynamic nature of attention and the possible harmful implications of attentional lapses presents a need for accessible, empirically validated methods, which can support day-to-day attentional functioning.

## Attention Enhancement and Restoration

In the field of attention enhancement, research has largely focused on network training approaches (i.e., structured practice with challenging attention-based tasks). Several reviews have been published in relation to these interventions. One in particular found robust enhancements to top–down aspects of attention following training with action video games (Bediou et al., 2017). Others looking at computerized cognitive training applications have identified a lack of evidence to support attention enhancement (Lampit et al., 2014). In addition, doubts have been raised in relation to whether any positive effects transfer to real world tasks beyond those studied in the lab (Butler et al., 2018; Harris et al., 2018). Importantly, the reviewed training practices involve repeat, structured practice, required over substantial time periods to be beneficial (Bediou et al., 2017). The relevance of this approach is questionable to someone struggling with a sudden dip in concentration levels or for those simply unable or unmotivated to commit to a training routine (Norris et al., 2018). Immediate forms of attention enhancement may be more accessible and better received than training programs.

In contrast to network training approaches, attention state training (AST) methods utilize state changes associated with experiences and practices that can optimize attentional performance (Tang and Posner, 2009; Posner et al., 2015). Of importance here is that these effects apply to a single exposure. Exposure to nature, for example, promotes a state of cognitive restoration where top–down control of attention, as a limited resource, can rest and recover from fatigue (Kaplan, 1995, 2001; Berman et al., 2008; Ohly et al., 2016; Stevenson et al., 2018). Physical exercise is associated with immediate boosts to attention from normal baseline levels (see Fernandes et al., 2018 for review); competing theories do exist, but emphasis has been placed on intermediary factors such as cerebral blood flow, arousal, and nervous system activation, which can facilitate attention performance after exercise (Gauthier et al., 2015; McMorris, 2016). Other state training approaches include mind–body practices such as yoga or mindfulness meditation. During practice, these techniques promote a higher state of focus on the present moment (Tang and Posner, 2009); evidenced through reduced and lasting brain activity in the default mode network (areas associated with mind-wandering) (Mason et al., 2007; Brewer et al., 2011; Fujino et al., 2018). Such approaches have proven beneficial when enhancing the restorative effects of nature (Lymeus et al., 2018) and providing short-term boosts to attention from typical baseline behavioral assessments (Gothe et al., 2013; Norris et al., 2018).

Evidence demonstrating the effectiveness of AST practices for attention, as well as broader aspects of physical, emotional, and cognitive well-being (Hartig et al., 2014; Good et al., 2016; Mandolesi et al., 2018), has given rise to their applied use in workplace and education settings. A growing emphasis is being placed on increased integration with nature (Raanaas et al., 2011; Lottrup et al., 2015; Moreno et al., 2018) and mindfulness, with mind–body practices implemented as workplace “breaks” to support cognition and well-being (Good et al., 2016). For these techniques to be applied more widely, however, there are physical, pragmatic, and motivational barriers that prevent a more comprehensive uptake of AST throughout daily life. For example, common barriers for meditation arise from a lack of knowledge about how to meditate and difficulties finding suitable times and spaces to practice (Hunt et al., 2020). The ability to spend time in nature is largely dictated by the physical locations where people live and work and is set to become increasingly difficult as urbanization continues. Critical to all forms of state training, the motivation or desire to integrate these practices is key. Despite the wealth of research evidence demonstrating the health benefits of exercise, this knowledge alone does not sufficiently motivate people to keep exercising (Duncan et al., 2010). We explore the potential of technologies considered “interactive” and “immersive” as tools to overcome some of these barriers, with a view to serve as regulatory solutions during critical attention-based tasks (e.g., demanding work assignments) or periods of low attention (e.g., cognitive fatigue).

## Interactive and Immersive Technologies

Interactive technologies are unique, as they encourage the user to modify the form and content of digital environments in real time (Steuer, 1992; Rubio-Tamayo et al., 2017). This is typical of challenge-based interactions found in digital games (Jin, 2012), certain mobile apps, and experimental setups designed for positive human functioning (Kitson et al., 2018; Niksirat et al., 2019). For the wider population, interactive experiences are most prominent within video games, ranging from simplistic, satisfying interactions in casual video games like *Bejeweled* (PopCap Games, 2001) or *Angry birds* (Rovio Entertainment, 2009), to more fast-paced, demanding interactions in action video games like *Call of Duty* (Infinity Ward, 2003) or *League of Legends* (Riot Games, 2009). Sandbox games such as *Minecraft* (Mojang Studios, 2011) go one step further, providing open-world access and the freedom to change virtual worlds at will. These interactions are already a routine aspect of daily life for many people, with both young and older adults searching for entertainment, mental stimulation, and stress relief (Whitbourne et al., 2013; AARP, 2019; The Entertainment Software Association, 2019). This includes gameplay on mobile devices during commutes, workplace breaks, and while waiting for appointments, with more involved gaming on purpose-built console systems and personal computers outside of work. Immersive technologies, on the other hand, offer both interactive and passive forms of content, but they are defined by the extent to which they deliver an inclusive, surrounding, and vivid illusion of reality to the senses of the user (Slater and Wilbur, 1997). Degrees of immersion are available in emerging mixed reality (MR) and augmented reality (AR) technologies, which merge virtual elements with our existing environment. Virtual reality (VR) goes one step further and replaces our environment with a synthetic one, achieved with the advent of stereoscopic displays, motion tracking hardware, and various input devices (Parisi, 2015). Previously, truly immersive experiences were limited to high end tethered systems such as the *HTC Vive* and the *Oculus Rift.* Now, with the advent of standalone VR systems such as the *Oculus Quest*, this level of immersion has become a more accessible experience and is expected to drive the frequency in usage of VR in the years to come (ARtillery, 2019).

Exploration into the state-inducing potential of interactive and immersive technologies (IITs) has already begun. A wealth of immediate positive state changes have been identified, including general states of well-being (Kitson et al., 2018), targeted mood states (Baños et al., 2012; Marin-Morales et al., 2018), stress relief (Russoniello et al., 2009; Rupp et al., 2017), mindfulness (Navarro-Haro et al., 2017; Sliwinski et al., 2017), physical exertion (Oh and Yang, 2010), pain relief (Kenney and Milling, 2016), and flow states (Jin, 2012; Hassan et al., 2020). Compared with traditional approaches, IITs offer greater access and potentially more efficient, enjoyable alternatives for state induction. Access is a common issue, for instance with meditative practices that often require expert guidance and privacy. Technology can facilitate this process, as seen with mobile apps affording novice meditators freedom to indulge in these practices in preferred settings (Mani et al., 2015). Digital games, interactive mobile apps, and VR have each been highlighted for their effectiveness in the cultivation of mindful states (Sliwinski et al., 2015, 2017; Navarro-Haro et al., 2017) and are particularly well-suited to meditating in busy environments (Salehzadeh Niksirat et al., 2017). For those in search of nature in built-up urban environments, we should expect to see the efficacy of digital nature interventions improve through more realistic and involved interpretations of nature afforded by immersive technologies (Schutte et al., 2017). Most virtual forms of nature exposure tested in restoration studies (2D images and videos) have produced comparatively lower effect sizes than real nature on measures of attention (Stevenson et al., 2018). In general, the added enjoyment and broader appeal of IITs might encourage a much larger integration of AST practices. Exergames, for example, utilize motion tracking in immersive and non-immersive video games, providing an innovative, highly enjoyable alternative for exercise (Oh and Yang, 2010; Moholdt et al., 2017).

It is not yet clear how these technologies might be applied to enhanced or restored states of attention. Much of the existing evidence relies on self-report or anecdotal evidence of greater focus during the activity; often involving experimental installations like *Sonic Cradle* (Vidyarthi, 2012), *Strata* (Du Plessis, 2017), and *Life Tree* (Patibanda et al., 2017), which apply immersive forms of nature and mindfulness practice. The literature is lacking a review of evidence utilizing pre- and post-behavioral performance measures of attention that can attest to the effectiveness and transference of IITs. A more general concern relates to the disruptive nature of technology devices. Smartphone and web applications, for example, are specifically designed to capture attention and hold it for long periods of time (Fogg, 2002; Lodge and Harrison, 2019). This combines with internally driven shifts in attention to these applications as we search for more immediately gratifying (unrelated) content (Wilmer et al., 2017). It is important to identify which devices and in what contexts this consumption of our attention might be beneficial to ongoing attentional functioning, rather than detrimental, as is feared (Ellison, 2012; Morin, 2013; Egan, 2016).

## Research Questions and Objectives

The current scoping review sets out to answer several key research questions from the existing literature: (1) what is the scope of existing literature concerned with the immediate effects of IITs on attention? (2) Which types of technological experiences have been investigated? (3) In what circumstances (if any) have these exposures been beneficial to attention? (4) What do these investigations currently tell us about the role of state changes for attention enhancement?

The core objective here is to identify current research gaps regarding the interventions and measures that have currently been used and develop an understanding of a state-induction approach to attention regulation and enhancement. This includes the role of the states outlined in the AST literature, as well as any other changes in state proven beneficial to attention. As the first review on the topic, this is an important step toward the identification and growth of potential interventions targeting low attentional performance or attention critical tasks.

## Methods

This study adopted a scoping review protocol based on the framework and recommendations proposed by Arksey and O’Malley (2005) and Levac et al. (2010), which includes five key phases: (1) outlining the research questions, (2) developing a search strategy, (3) selecting relevant studies, (4) charting the data, and (5) collating, summarizing, and reporting the results. A scoping review was chosen over other review methods, as they are well suited to explorative investigations where there have been no comprehensive reviews in the area already (Fulop, 2001). The main aim of this review process is to map out the extent of existing literature while identifying research gaps and making recommendations for future research (Booth, 2016). As with systematic reviews, scoping reviews contain a high degree of rigor, but the extent to which they include a formal quality assessment of studies depends on the purpose of the review itself (Armstrong et al., 2011). To help guide future research in the field, a broad review of evidence quality was applied using the (Australian) National Health and Medical Research Council levels of evidence framework (NHMRC, 2009). Additional factors that determine the significance and relevance of the reviewed evidence are also assessed, including the use of comparative interventions, the durations of each intervention, and the various measures used (including attention and any broader physiological, neurological, cognitive, or subjective well-being measures).

### Study Search

A systematic search of three academic databases was conducted: MEDLINE, Embase, and PsycINFO. This included all studies up to November 2018. The first part of the search was focused on immersive and/or interactive technologies in accordance with the previously given definitions. The second part included a broad range of terms related to the many aspects of attention:

*(VR OR “virtual realit^∗^” OR “video gam^∗^” OR videogam^∗^ OR “computer gam^∗^” OR Exergam^∗^ OR App OR Ipad OR Wii OR Kinect OR Tablet OR “mobile game^∗^” OR HMD OR “head mounted display” OR “role playing gam^∗^” OR “mixed realit^∗^” OR “augmented realit^∗^” OR “digital gam^∗^” OR “serious gam^∗^” OR Gamification OR “brain train^∗^”)* ***AND*** *(“Mind wandering” OR “Attentional scope” OR “Attention regulation” OR “Attention restoration” OR Restorat^∗^ OR “Attentional blink” OR “Selective attention” OR “Sustained attention” OR “Executive attention” OR “Divided attention” OR “Attention network” OR “Attentional blindness” OR “Inattentional blindness” OR “Spatial attention” OR “exogenous attention” OR “endogenous attention” OR “Cognitive control” OR vigilance)*

Reviews that were identified as being relevant to the current review and the state training approach to attention enhancement were manually searched for additional records. This involved searching reference lists and tables of included studies in a series of reviews: the use of interactive technologies for mindfulness (Sliwinski et al., 2017), immersive interactive technologies for positive change (Kitson et al., 2018), attention restoration theory (Ohly et al., 2016; Stevenson et al., 2018), exercise and attention (Fernandes et al., 2018), exergaming and cognition (Ogawa et al., 2016), videogames and cognition (Bediou et al., 2017; Sala et al., 2017), and computerized cognitive training devices (Lampit et al., 2014; Harris et al., 2018). A “snowball” technique was also adopted, which involved searching through citations within articles when they appeared relevant to the review (Pham et al., 2014).

### Inclusion–Exclusion Criteria

Specific inclusion–exclusion criteria were implemented to ensure that reviewed studies were applicable to the general public and reflective of immediate changes in attention. Primarily, studies were required to involve a single session using an interactive or immersive technology. Circumstances where technology exposures were repeated over several sessions or utilized attention network training methods (e.g., brain training games, cognitive training apps) were not included.

The definitions outlined in the *Introduction* were used to guide exclusion of non-interactive and non-immersive technologies. Interactive technologies were characterized by the ability to directly influence the elements within digital environments in real time; similarly to Kitson et al. (2018), technological experiences that afford only a minor degree of influence such as web pages, social media use, video instructions, and guided mobile apps were excluded as non-interactive. Technologies were considered immersive by their physical properties, by which they created an extensive and surrounding illusion of reality to the senses. This applies to VR, AR, and MR technologies primarily.

The remaining criteria included the following:

• Only peer-reviewed original research studies—all reviews, conference papers, and dissertation/theses were excluded.
• Participants were all healthy adults (18+)—no children or participants diagnosed with impairments, illnesses, or at-risk factors associated with cognitive functioning. Discussions concerning the developmental aspects of attention or attentional changes with respect to attention-compromised groups are beyond the scope of the review.
• Technology use was followed by a direct assessment of top–down attention compared with baseline measures or control conditions—studies that did not include a baseline measure of attention but compared postintervention measures with other interventions were also included in the review.
• Studies used one or more behavioral performance measure of attention—studies that only used other methods (e.g., EEG, self-reports) were excluded.

### Charting the Data

Table 1 provides a complete summary of the study characteristics from the final reviewed articles. The following variables were extracted from the final pool of studies to answer the research questions:

**TABLE 1:** Charted data for each of the final reviewed studies.

Paper	IIT interventions	Comparative interventions	Duration	Participants	Attention assessment	Positive effects on attention	Additional outcomes	Level of evidence
Aliyari et al. (2015)	Multiplayer football **video game** (FIFA 2015) played on an Xbox 360 console in a single knockout competition.	N/A	45 min	University students, *n* = 32, *m* = 20 years	PASAT	N/A	No attention changes. Decrease in stress (salivary cortisol concentration).	IV
Barcelos et al. (2015)	Two different versions of a **VR bike tour:** high cognitive load (including gameplay components) or low cognitive load (just biking). Both participant groups asked to maintain target heart rates calculated using the Karvonen equation.	N/A	20 min	Older adults, *n* = 64, *m* = 82 years	CTT, Color Stroop	Improved attention with most improvement in the high cognitive challenge condition (CTT).	No change in Stroop scores for either group.	II
Cassarino et al. (2019)	**VR driving simulator** in a rural (nature) setting or an urban setting.	N/A	10 min	University students, *n* = 38, *m* = 22 years	SART	N/A	No attention changes.	II
Engelhardt et al. (2015)	One of four different versions of **video game** “Doom” played on a desktop computer: violent easy, violent hard, non-violent easy, non-violent hard.	N/A	15 min	University students, *n* = 238, *m* = 19 years	Spatial Stroop		Challenging game version when experienced as difficult associated with impaired attention compared to easy versions of the game (no comparison with baseline).	II
Hwang and Lu (2018)	One of four fighting video game versions: sedentary narrative, sedentary no-narrative, active narrative and active no narrative. Active (**exergame**) version was Kung-Fu Kinect with the Xbox-One and Kinect sensor, sedentary (**video game**) version was Street Fighter with PlayStation 4.	N/A	30 min	Young adults, *n* = 100, *m* = 21 years	Psychomotor vigilance task	N/A	No attention changes. Improved working memory performance correlated with increased heart rate from exergame conditions (delayed match-to-sample memory task). Both exergame versions involved moderate-vigorous levels of exercise intensity, but this was higher in the narrative version (HR, perceived exertion).	II
Kirk et al. (2013)	**Exergaming** with a combination of 9 activities from Wii sports and Wii fit.	N/A	21 min	Older adults, *n* = 20, *m* = 61 years	Trail task B	N/A	No attention changes. Overall improvement in mood (PANAS) and a light–moderate exercise intensity (HR).	IV
Kozhevnikov et al. (2018) (1)	First person shooter **video game** (Unreal Tournament) on a desktop computer. Game difficulty was regularly adjusted in accordance with player’s skill level to achieve optimal challenge-skill balance.	Passive observation of game playing.	30 min	Experienced video game players, *n* = 32, *m* = 21 years	ABT	Improved attention immediately and 30 min after intervention compared to baseline. Watchers did not show any increase.	Higher reports of flow experiences amongst those who played the game (FSS, interview questions). FSS directly correlated with improved ABT performance.	II
Kozhevnikov et al. (2018) (2)	One of three different versions of Unreal tournament: underchallenge (easy), optimal-challenge (balanced) and overchallenge (very hard).	N/A	30 min	Casual video gamers *n* = 56	ABT	Optimally challenged participants exhibited improved attention immediately and 30 min after	No attention changes for under-challenge or over challenge groups. Only the optimal challenge group had significant decreases in parasympathetic activity (EKG). Increased sympathetic activity in both optimally challenged and over challenged groups.	II
Kozhevnikov et al. (2018) (3)	Same as experiment 1.	N/A	30 min	Experienced video game players, *n* = 23, *m* = 21 years	ANT (alerting, executive, orienting)	Improved alerting attention	No executive or orienting attention changes. Improved visual memory and spatial transformation capacities (VMT, MRT).	IV
O’Leary et al. (2011)	Either **exergaming** (Wii fit) or **video gaming** (Mario kart) using the Nintendo Wii motion controls. The exergame intervention involved a moderately intense level of exercise.	Seated rest or aerobic exercise of the same intensity as the exergame intervention (treadmill walking).	20 min	University students, *n* = 32, *m* = 21 years	Flanker task	N/A	No changes in attention from video game or exergame interventions. Aerobic exercise facilitated improved attention and enhanced neuroelectric indices underlying attentional resource allocation (EEG). Same HR increase for the aerobic exercise and exergame conditions (HR).	III-1
Qiu et al. (2018)	Multiplayer action strategy **video game** (League of Legends) played on a computer.	N/A	1 h	Experts and non-experts in the video game intervention, *n* = 29, *m* = 23 years	UFOV	Both experts and non-experts exhibited improved attention.	Different arousal responses for experts and non-experts evidenced by EEG: increased N2 amplitude for experts but not non-experts, decreased P3 amplitude for experts but no difference for non-experts.	IIV
Rupp et al. (2017)	**Casual video game** ‘Sushi Cat 2’ on a computer following cognitive fatigue induction	Passive break or guided relaxation.	5.5 min	University students, *n* = 66, *m* = 20 years	Digit-span backward task	N/A	No attention changes from video game intervention. Improved attention from guided relaxation. Improved positive mood in all 5 factors of the PANAS from casual video game (PANAS).	II
Valtchanov et al. (2010)	Explorative **VR** nature environment or a series of abstract paintings viewed in VR.	N/A	10 min	University students *n* = 22; age range, 17–26	Maths quiz	N/A	No attention changes. Improved positive mood and reduced stress in the VR nature intervention (SCL, HR, ZIPERS).	III

*PASAT, Paced Auditory Serial Addition Test; CTT, Color Trails Test; SART, Sustained Attention to Response Task; ABT, Attentional Blink Task; ANT, Attention Network Task; UFOV, Useful Field of View Task; HR, heart rate, PANAS, Positive and Negative Affect Schedule; VMT, Visual Memory Test; MRT, Mental Rotation Test; ZIPERS, Zuckerman Inventory of Personal Reactions; SCL, skin-conductance level.*

1. IIT intervention—the name and type of technologies used, including experimental manipulations, with descriptions of the involvement by the participants.
2. Comparative intervention—alternate activities used to compare against IITs.
3. Duration—of each intervention.
4. Participants—age, number, and group membership (e.g., student, gamer, etc.).
5. Attention assessment—the behavioral performance measures of attention used.
6. Positive effects on attention—if any were found and in what circumstances.
7. Additional outcomes—where no or negative effects on attention were found or additional measures were used relevant to changes in participant’s state.
8. Level of evidence—categorized according to NHMRC (2009) evidence hierarchy guidelines: level I (systematic reviews of level II studies), level II (randomized controlled trial studies), level III-1 (pseudorandomized controlled trials) level III-2 (comparative studies with concurrent controls), level III-3 (comparative studies without concurrent controls), level IV (case series with either posttest or pretest/posttest outcomes).

## Results

**FIGURE 1:**
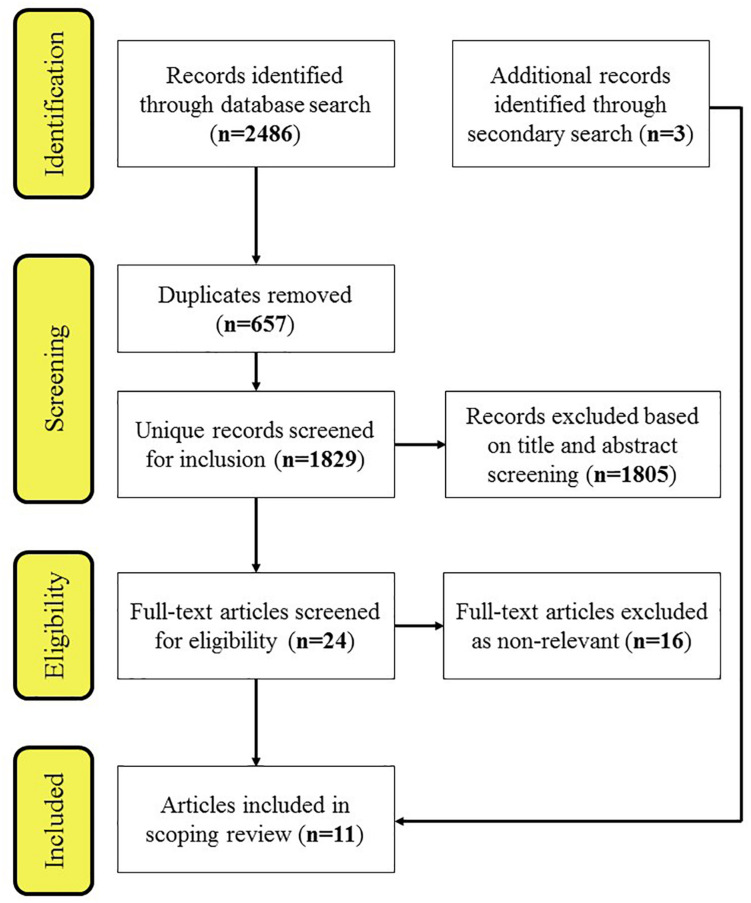
Study search process including (a) initial identification, (b) screening based on title and abstracts, (c) checking full-text articles against inclusion criteria, and (d) the final reviewed papers.

A total of 11 papers containing 13 experiments were included for final analysis in the review. The process from article identification to final inclusion is represented in Figure 1.

### Technology Intervention Characteristics

A complete description of the intervention characteristics, including the device, the game/experience type, and the intervention duration for each experiment can be seen in Table 1. Seven of the final articles measured changes in attentional performance following the use of commercially available video games. Video games are by far the most popular, widely available interactive technology and thus have received the most attention concerning the effects on cognition. Two of the studies modified the content of the video games to produce different levels of experienced difficulty (Engelhardt et al., 2015; Kozhevnikov et al., 2018). One study purposefully included or excluded a story narrative at the beginning of the game to change participant’s level of engagement (Hwang and Lu, 2018). The other four studies used only the original versions of the video games, as they are commercially available (O’Leary et al., 2011; Aliyari et al., 2015; Rupp et al., 2017; Qiu et al., 2018). Four of the reviewed articles used exergames: defined as video games that utilize motion tracking and require physical exertion to play (Oh and Yang, 2010). One of the exergames was a movement-based fighting game acting as a comparison for a sedentary fighting video game (Hwang and Lu, 2018). Three of these were commercially available exergames played using the Nintendo Wii and the Xbox Kinect (Rupp et al., 2017). One of the studies used a non-commercial VR-enhanced bike tour used with and without challenging gameplay elements (Barcelos et al., 2015). The two remaining interventions also utilized VR; these included a VR driving simulation (Cassarino et al., 2019) and a restorative nature experience explored while wearing a head mounted display (Valtchanov et al., 2010). Use of the technologies ranged in durations from 5.5 min to 1 h.

### Attention Measures

A wide variety of behavioral attention measures were employed. Just two of the reported measures were used by more than one study, albeit with different versions: color/letter versions of the Trails task (Kirk et al., 2013; Barcelos et al., 2015) and spatial/color versions of the Stroop task (Barcelos et al., 2015; Engelhardt et al., 2015). The Flanker task, executive component of the Attention Network Task (ANT), Trails task, and the Stroop task are considered measures of executive attention. The Sustained Attention to Response Task (SART), Paced Auditory Serial Addition Test (PASAT), and the psychomotor vigilance task are considered measures of sustained attention. The remaining measures assess degrees of top–down attention but also broader areas of cognitive ability. These include the Attentional Blink Task (ABT), Useful Field of View (UFOV), orienting and alerting components of the ANT, digit-span backward task, and a maths quiz. The ABT, for example, has been closely linked with executive attention deficits (Marois and Ivanoff, 2005; Hommel et al., 2006), yet multiple factors such as working memory, episodic registration, response selection, distractor inhibition, and a generalized limit of attentional resources have also been proposed as responsible for attentional blink deficits (Shapiro et al., 1997; Dux and Marois, 2009).

### Nature of the Effects

Results were judged as “positive” if there was a significant increase in attention scores pre–postintervention. Out of the 13 experiments, there were five positive effects on attention, although two of these used additional attention measures where positive effects were not found (Barcelos et al., 2015; Kozhevnikov et al., 2018). From the studies reporting positive effects, only Kozhevnikov et al. (2018) used a comparative intervention that can attest to the significance of the findings against non-interactive/immersive interventions (i.e., playing the video game vs. passive observation). The remaining studies reporting positive effects had either no comparative intervention or used just a slightly modified form of the IIT intervention as a control. The remaining studies found no changes in attention. There was one case where a negative effect on attention was reported, however, this was based on a comparison between conditions without baseline attention measures; it is not clear whether attention performance worsened compared to participant’s normal attention ability (Engelhardt et al., 2015). To explore in what circumstances IITs were beneficial to attention, the results are outlined according to the interventions and observed relationships between attention and other state-based changes. These include physical exertion, cognitive challenge, arousal, and mood/stress.

#### Physical Exertion and Attention

The primary case examining physical exertion in relation to improved attention was an immersive aerobic exercise intervention (Barcelos et al., 2015). The intensity of the exercise was not reported as an outcome measure, yet all groups were actively encouraged to maintain a target heart rate throughout the intervention. In this case, 20 min of immersive bicycle riding was associated with improved executive attention. Interestingly, Hwang and Lu (2018) found a positive correlation between exercise intensity levels and working memory performance following moderate–vigorous exercise within an exergame. However, there was no impact on the measure of sustained attention. The remaining studies of physical exertion involved low–moderate physical exertion levels using exergames, which did not correspond with any attention gains (O’Leary et al., 2011; Kirk et al., 2013). Of interest, O’Leary et al. (2011) found improvements in executive attention following an aerobic exercise intervention but not for an exergaming intervention (both of which involved moderate levels of exercise intensity).

#### Cognitive Challenge and Attention

Three studies manipulated the cognitive challenge imposed by IIT interventions to explore the impact on attentional functioning. These used a combination of different approaches: comparing an immersive intervention with and without a challenging gameplay component (Barcelos et al., 2015), comparing video games set at different difficulty levels between groups (Engelhardt et al., 2015; Kozhevnikov et al., 2018), and one case where game difficulty was continuously adjusted to match the skill and performance levels of individual participants (Kozhevnikov et al., 2018). Attentional improvements related to an immersive bicycle tour were greater with the addition of a challenging gameplay component (Barcelos et al., 2015). Engelhardt et al. (2015) found that high task difficulty (where a video game intervention is experienced as overly difficult) was associated with worse executive attention. Kozhevnikov et al. (2018) found attention enhancements for those in an optimally challenged condition, with no improvements for under- and overchallenged groups. This was the only study to examine these effects in relation to the flow state. “Flow” refers to a state of optimal experience where people are completely absorbed in a challenging task, to the point of forgetting time, fatigue, and virtually everything else but the activity itself (Csikszentmihalyi, 2014). Dimensions of flow including “loss of self-consciousness” and “concentration on task” from the video game intervention were directly correlated with improvements on an attentional blink task.

#### Arousal and Attention

Several studies recorded changes in arousal resulting from sedentary videogame interventions. Arousal changes were reflective of an increased level of engagement presented by challenging video game interventions (as opposed to physical exertion). The role of arousal for attention appeared mixed. Kozhevnikov et al. (2018) reported a direct association between arousal and the enhanced attentional states resulting from the challenging video game intervention. On the other hand, Qiu et al. (2018) found improved UFOV performance following a video game intervention but an inconsistent relationship with arousal responses.

#### Mood/Stress and Attention

Four of the reviewed studies included measures of mood alongside attention measures. These included an immersive nature experience, a casual video game, an exergame, and a competitive video game (Valtchanov et al., 2010; Kirk et al., 2013; Aliyari et al., 2015; Rupp et al., 2017). Each of these were associated with improvements in mood and/or reduced stress but no corresponding changes in attention.

## Discussion

The current review provides the first attempt to unify a broad range of investigations by a common theme: immediate changes in attention from interactive or immersive experiences not associated with practice or training effects. We have identified a limited and largely unsupportive literature base in this regard, with most studies reporting no effects on attention. The relevance of this finding is discussed considering a lack of studies and IIT interventions applying AST methods of attention enhancement. The positive effects reported suggest that certain video games and immersive exergames can provide immediate temporary boosts to attention in healthy adults beyond normal baseline levels, effects that would commonly be associated with traditional forms of meditation or exercise (Tang and Posner, 2009; Posner et al., 2015). The tailored approach to cognitive challenge afforded by these interventions is discussed as a key factor.

### Cognitive Challenge, Flow, and Attention

The cognitive challenge imposed by IITs was the most influential factor that determined changes in attention. This was evident with the attentional gains for participants who were “optimally challenged,” compared with those presented with tasks where the difficulty levels were too high or too low (Barcelos et al., 2015; Engelhardt et al., 2015; Kozhevnikov et al., 2018). The enhanced attentional states reported have direct relevance to the flow state, which can occur when one’s skill levels are well matched with the challenges demanded by a task. Flow has been described as a state of “effortless attention” (Bruya, 2010) and a state of complete focus in a task, at the expense of all other internal and external distractors (Csikszentmihalyi et al., 2005). Kozhevnikov et al. (2018) adopted a critical approach to delineate the role of flow, which included a validation of the intervention regarding the state changes involved and a manipulation of the intervention to investigate the importance of optimal challenge compared with under- and overchallenged versions of the same task. Flow was directly related to the attentional gains from the video game intervention. Where overly challenging tasks are deemed to be a drain on cognitive resources (Engelhardt et al., 2015), optimally challenging tasks may present an avenue for greater cognitive capacity. Questions remain regarding the effects across a broader range of attentional components and the role of other factors such as arousal states (Hwang and Lu, 2018; Qiu et al., 2018).

Flow is a state not yet recognized within the AST literature. This may in part be due to the lack of evidence regarding its lasting effects on attention; previously, emphasis has been placed on the role of attention for obtaining and sustaining flow states (Nakamura and Csikszentmihalyi, 2002). Central to the concept of AST is that an activity can produce changes in the mind–body state that benefit attentional functioning in everyday tasks. Although preliminary, evidence reviewed here demonstrates that video games can produce flow-like states, which can enhance performance on behavioral measures of attention, effects lasting 30 min after the intervention (Kozhevnikov et al., 2018). Where video games have predominantly been used as a network training intervention, focus on their state effects may support a use case more applicable to AST. Video games are highly regarded for their flow-inducing potential because they allow precise matching of challenge levels as well as clear goal setting and direct feedback (Bruya, 2010). These qualities extend to immersive technologies as well, with the added advantage of removing external distractions (Brondi et al., 2015). The reviewed studies have demonstrated the use of IITs in a way that allows a direct comparison between interventions based purely on the cognitive challenge they present. This presents a valuable tool to learn more about the influence of cognitive challenge and potentially offer experiences designed to facilitate attentional functioning through flow state induction.

### Application of Attention State Training Practices

The acknowledgment of a collective range of mind–body states associated with attentional functioning was founded in the AST literature (Tang and Posner, 2009; Posner et al., 2015). That includes state changes resulting from meditative practices, exposure to nature, and physical exercise. To date, very few studies have explored the potential of these practices for attention regulation when applied within IITs. A search for the term *“attention state training”* within the reviewed articles produced zero results, which suggests a lack of research aims centered on contributing to the AST literature. Most notably, there were no implementations of meditative or mind–body practices in the reviewed studies. This is not overly surprising considering the preliminary nature of research findings related to cognitive enhancement from brief meditation sessions (Mrazek et al., 2012; Colzato et al., 2015; Lymeus et al., 2018; Norris et al., 2018). These effects are normally associated with regular training over a matter of weeks or months (Jha et al., 2007; MacLean et al., 2010; Elliott et al., 2014; Lippelt et al., 2014). One recent study found improved executive attention after only 5 days training with an interactive mindfulness mobile application (Salehzadeh Niksirat et al., 2017). This shows promise for the role of meditation in IITs to facilitate attentional functioning, yet the effectiveness of such interventions without training awaits further validation.

The most prevalent application of AST practices here was exercise, evident within studies looking at the role of exergame interventions. With traditional types of exercise, enhanced attention is most closely associated with moderate and above levels of exercise intensity and relates most prominently to attention tasks that involve executive functioning (Córdova et al., 2009; Kamijo et al., 2009). This was partly reflected in the reviewed studies, with executive attention and working memory performance tied to short bouts of moderate–vigorous levels of exercise activity (Barcelos et al., 2015; Hwang and Lu, 2018). However, physical exertion was clearly not a guarantee of any attentional performance gains ascertained by exergame activities (O’Leary et al., 2011), and other components related to the cognitive challenge presented by the interventions may prove critical (Barcelos et al., 2015). Exergames present a highly enjoyable alternative for exercise (Oh and Yang, 2010; Moholdt et al., 2017) in which physical activity and cognitive demands can be tailored to each individual. This makes them the ideal vehicle for future studies to examine these factors further and promote interactions that will be beneficial to cognitive functioning.

Two studies assessed the role of immersive/interactive nature for the purpose of attention restoration, but in both cases, the interventions had no impact on attention (Valtchanov et al., 2010; Cassarino et al., 2019). Critically, both studies contained methodological limitations related to the attention measures used (a maths quiz), the choice of fatigue-inducing activity (or lack of), and the fatigue inducing nature of the restorative environment (a driving task). These elements would normally contradict recommendations for studies of attention restoration (Stevenson et al., 2018). This lack in suitable investigatory evidence from restoration studies using immersive forms of digital nature may contribute to the lower effect sizes currently attributed to digital nature interventions (Stevenson et al., 2018). A third study aimed to explore if casual video games could restore attentional functions following fatigue, but this was not successful (Rupp et al., 2017). These studies represent the beginning of a search for accessible interventions, which can take advantage of the qualities of IITs and place users in restorative states of mind. To the date that the current literature search was carried out, evidence for attention restoration from these technologies is restricted to ratings of perceived attention restoration (Schutte et al., 2017), and awaits further validation using behavioral performance measures to build on the evidence base from digital nature exposures (Berman et al., 2008; Gamble et al., 2014).

### Limitations and Future Research

To identify the scope of the current literature and identify any beneficial effects on attention, we purposefully included a broad range of IIT interventions. That includes both experimental installations and commercially available experiences, all created for different purposes (e.g., entertainment, exercise, challenge, well-being, driving simulation, etc.) and containing vastly different user experiences. This prevents a meaningful comparison of effect sizes at this stage. Out of the final 13 experiments, seven were considered randomized controlled trials according to the Cochrane criteria (Higgins and Green, 2011), and only three included a comparative intervention. This reflects on the relevance of the findings; without a control, we cannot be sure that the positive effects would not have been obtained without treatment (Kozhevnikov et al., 2018^(3)^; Qiu et al., 2018). Without comparative, established interventions, assumptions about the potency of IITs remain unvalidated (Barcelos et al., 2015; Kozhevnikov et al., 2018^(2)^). The exclusion of broader areas of cognitive functioning with likely implications for top–down attention is another possible limitation of the review. Working memory, for example, is considered tightly linked with executive attention (Diamond, 2013; Engle, 2018), and studies have shown both positive and negative effects on working memory performance following video game and exergame interventions (Kuschpel et al., 2015; Hwang and Lu, 2018).

We hope to encourage further research in this field that can attest to the effectiveness of IITs for attention restoration and enhancement. As an ideal standard, researchers should aim to implement randomized controlled trials with pre–postattention measures and appropriate comparative interventions. A combination of different measures is advised considering the disparity between outcomes, even between measures of similar attention components (Barcelos et al., 2015). We recommend, where possible, utilizing established forms of attention enhancement as comparisons. This was the case with two of the reviewed studies: comparing exergames with treadmill exercise (O’Leary et al., 2011) and casual video games with guided relaxation (Rupp et al., 2017). Future studies might also compare immersive digital nature with actual nature (Berman et al., 2008; Schutte et al., 2017) or interactive with guided forms of meditation (Colzato et al., 2015; Salehzadeh Niksirat et al., 2017) for example. In some cases, alternate methods considered established and applicable to laboratory settings do not exist. Flow, for instance, is a widely reported phenomena (throughout sport, work, and creative activities) (Csikszentmihalyi, 2014), yet notoriously difficult to induce in experimental settings before the applied use of video games (Moller et al., 2010). Researchers should also compare against typical task-unrelated break activities, such as the physical, social, and cognitive microbreaks taken in the workplace (Kim et al., 2017). This would contribute to the relevance of any findings, aiming to promote well-informed break choices. While exploring effectiveness, it is also important to consider the suitability of IIT interventions to daily life. The time required to reach improved attentional states and the following duration of those effects is a critical factor. Just one of the reviewed studies assessed the duration of attention enhancement (Kozhevnikov et al., 2018). What is more, the duration of the interventions ranged between 5.5 min and 1 h; researchers should prioritize smaller intervention durations applicable to busy daily routines. Considering the broader physical, pragmatic, and motivational barriers facing AST, the use of longitudinal mixed method studies integrating IIT interventions within workplace and education settings is also strongly encouraged (Taylor et al., 2013; Moreno et al., 2018).

As a collective aim, research investigations should be looking at ways in which IITs can support day-to-day attentional functioning and mental health. The continued use of broader well-being assessments is encouraged to explore the extent of their potential health benefits (Valtchanov et al., 2010; Kirk et al., 2013; Aliyari et al., 2015; Rupp et al., 2017; Kitson et al., 2018). Furthermore, using state-based measures alongside cognitive assessments will ascertain the important driving factors of attention enhancement and inform the development of future applications. In accordance with the attention-related states identified in the review, this should include state measures of mindfulness (Tanay and Bernstein, 2013; Fujino et al., 2018), restoration (Pasini et al., 2014; Basu et al., 2019), flow/challenge (Jackson and Marsh, 1996; Engelhardt et al., 2015), arousal (Kozhevnikov et al., 2018; Qiu et al., 2018), and physical exertion (Sylvia et al., 2014).

## Conclusion

Considering the emergence of technological applications promising immediate focus or concentration-enhancing properties (Ramey, 2017), the current literature is lacking a substantial research focus that can support the role of IITs in this regard. To date, the potential for lasting improvements gained from computerized training has taken clear precedence, evident in the growth of network training intervention studies regarding cognitive training tasks and video game training (Bediou et al., 2017; Tetlow and Edwards, 2017; Harris et al., 2018). With AST practices, there is a clear gap in evidence related to meditation and restorative environments; this is most likely due to a lack of suitable technology applications available and research interest at this stage. Still, in certain circumstances, interactive and immersive applications built for entertainment purposes can produce enhanced attentional states in healthy adults via mechanisms related to cognitive challenge and flow. Where short-term boosts in attention have been disregarded as of little practical use (Bediou et al., 2017), the current review acknowledges their significance for attention enhancement during critical periods. Perhaps more importantly, this approach holds a significant advantage over network training methods due to the wide-spreading benefits for health and well-being (Kitson et al., 2018). Even with no positive effects on attention, those studies found positive effects on mood, physical exertion, and engagement, which provides further motivation for their use in future research and day-to-day life.

## Author Contributions

AB, JS, and LB contributed to the initial conceptualization and planning of the scoping review. AB identified and screened relevant articles, performed a qualitative synthesis of the data, and wrote the first draft of the manuscript. All authors contributed to manuscript revision and read and approved the submitted version.

## Conflict of Interest

AB is employed part-time by virtual reality software company Liminal VR. LB has a funded collaboration with the virtual reality software company Liminal VR. The remaining author declares that the research was conducted in the absence of any commercial or financial relationships that could be construed as a potential conflict of interest.
